# Supplementary material for: “You could find a good or a bad provider, I would say you just have to go and see”: A qualitative study of the influence of perceptions of service quality on family planning service use in Burkina Faso
Source: PLOS Glob Public Health. 2023 Mar 31;3(3):e0001780. doi: 10.1371/journal.pgph.0001780 (PMC10065260; doi:10.1371/journal.pgph.0001780)
Supplement: Attachment — Submitted filename: Response to reviewer_Influence_3_10_23.docx [file pgph.0001780.s001.docx]

Response to reviewer:

1. (as written by reviewer): You mention that community leaders were involved in identifying women to participate in FGDs, who were these leaders? Mostly men? Women? Religious leaders? Any chance they could have influenced the sample unduly?

(as rewritten by editor): Provide more information on the types of community leaders involved in identifying women to participate in FGDs and how you ensured that this did not lead to biases in the sample or indeed any undue coercion.

Response: Thank you for this request for clarifications on who we engaged to help identify participants in the study. We have added additional details on this in the text on page 7. We have clarified that community leaders were women and men who run community organizations or associations and were knowledgeable about the realities and needs of people in their communities. We clarify that we did not give these community leaders details of the specific subject of the interviews to avoid biasing who they proposed as participants and participants had the right to accept or refuse participation.

2. (as written by reviewer): There was mention of a small number of FGDs with men, but this is not in the FGD table nor the findings referenced elsewhere that I could see. If these FGDs were not included in the analysis maybe just remove this sentence?

(as rewritten by editor): Please address the issue of a  small number of FGDs conducted with men and provide a table and details on this as necessary, and explain whether those were included in the analysis.

Response: In this paper, we do not include the men’s focus group discussion information and thus, as suggested by the reviewer, we have removed the mention of them in the text.

3. (as written by reviewer): A bit of context as to why the two sites of Bobo & Banfora were selected would be helpful. These are both cities, yet you say the results are not generalisable to other cities like Ouaga, why not?
   (as rewritten by editor): Please provide context and procedure around the selection of the 2 study urban sites of Bobo & Banfora. Please explain why you state that the results are not generalisable to other cities or urban sites.

Response: We have now added greater details in the text (see page 6) on the selection of the two study cities Bobo Dioulasso and Banfora. This selection was related to a partner project called Beyond Bias (<https://www.pathfinder.org/projects/beyond-bias/>) that was seeking to improve health care service quality, especially for young people. These two cities were places the project worked and we recruited participants in communities near facilities where the program worked and in communities near facilities where the program did not work. Since we did not find distinctions by Beyond Bias engagement, this is not discussed in the text. That said, because of the connection to the Beyond Bias project, the results from these two cities may not be generalizable to other cities where there was no Beyond bias program. This has been clarified in the limitations as well.
